# The ‘Botanical Triad’: The Presence of Insectary Plants Enhances Natural Enemy Abundance on Trap Crop Plants in an Organic Cabbage Agro-Ecosystem

**DOI:** 10.3390/insects10060181

**Published:** 2019-06-22

**Authors:** Binita Shrestha, Deborah L. Finke, Jaime C. Piñero

**Affiliations:** 1 Division of Plant Sciences, University of Missouri, Columbia, MO 65211, USA; b.shrestha@ufl.edu (B.S.); finked@missouri.edu (D.L.F.); 2 Stockbridge School of Agriculture, University of Massachusetts, Amherst, MA 01003, USA

**Keywords:** habitat manipulation, botanical triad, integrated pest management, sustainable agriculture, parasitism

## Abstract

Habitat manipulation through the incorporation of non-crop plants such as trap crops (to lure pests away from the cash crop) and insectary plants (to provide resources for natural enemies) into agro-ecosystems is an ecological approach to pest management. In a field-scale study, we quantified the effects of integrating the use of trap crops with insectary plants as a novel method to control pest herbivores in an organic cabbage agro-ecosystem. We hypothesized that pests would be concentrated in the trap crop habitat and suppressed by insectary-subsidized natural enemies in situ. We documented arthropod abundance (both adults and immature stages) associated with (1) two insectary plant species (sweet alyssum, *Lobularia maritima*, and buckwheat, *Fagopyrum esculentum*) either alone or in combination; (2) a trap crop mixture of mighty mustard (*Brassica juncea*), red Russian kale (*Brassica* *oleracea* var. acephala), and glossy collards (*Brassica oleracea* var. italica), and (3) cabbage cash crop (*Brassica oleracea* var. capitata). Trap crops were more attractive to pests than the cash crop. On a per-plant basis, densities of the herbivores *Evergestis rimosalis*, *Trichoplusia ni*, and *Plutella xylostella* were 154, 37, and 161× greater on the kale trap crop than on the cabbage cash crop, and 54, 18, and 89× greater on the collards trap crop than on the cash crop. Insectary plants contributed to the consumption of pests that aggregated on the trap crop. Parasitism of *E. rimosalis* by the braconid wasp *Cotesia orobenae* was significantly increased, and the abundance of eggs and larvae of the predatory coccinellid beetle *Coleomegilla maculata* was greater on the trap crop in the presence of insectary plants compared to trap crops that lacked insectary plants. The ‘Botanical Triad’ of cash crop, trap crop, and insectary plants represents a new type of agro-ecosystem manipulation that integrates ecosystem service providers (e.g., predators and parasitoids) within the cropping system.

## 1. Introduction

Modern agriculture practices such as monoculture production, intensive land use, tillage, and chemically-based pest control have contributed to the loss of biodiversity in many agricultural production areas, negatively affecting functional biodiversity and ecosystem services such as pollination and biological pest control [1,2,3,4]. In agricultural monocultures, non-crop vegetation is usually excluded from both cropped areas and adjacent fields, leading to simple landscapes that are dominated by few or single plant species [4,5]. Less diversified crop production systems have often led to more pest outbreaks compared to more diversified systems, resulting in increased pesticide use [6,7]. The combined effects of landscape simplification, increased pest pressure, and greater pesticide use have contributed to the rapid loss of biodiversity in agroecosystems, which greatly affects the functioning of natural pest control [7,8,9].

Global interest in ecological intensification [10,11], and, more specifically, on sustainable pest management, has reinforced the importance of biodiversity conservation in agro-ecosystems [12]. From a pest management perspective, boosting populations of naturally occurring enemies of arthropod pests via habitat diversification has been advocated as a more sustainable alternative to chemically-based pest control [2,13,14,15,16]. Several studies have demonstrated that the establishment of non-crop habitats around field margins can provide ecosystem services, including enhanced biological pest control [17,18,19,20]. Natural enemies are often more diverse and abundant in landscapes containing large numbers of natural or semi-natural habitats than in structurally simple, intensely cultivated landscapes. For instance, one meta-analysis of 46 landscape-level studies found that natural enemies have a strong positive response to landscape complexity [20].

Trap cropping is one habitat diversification tactic that can be used to attract, divert, intercept, or retain targeted pests in non-crop habitats to protect economically valuable crop plants [2,21,22,23]. To be effective, trap-cropping systems must congregate and retain the pest on trap crop plants, thereby reducing the pest populations in the cash crop [23,24,25]. In the Brassica agro-ecosystem, Indian mustard and glossy collards have been evaluated successfully as trap crops to control the diamondback moth (*Plutella xylostella* [L.]) (Lepidoptera: Plutellidae) [26,27,28]. However, most studies have focused on individual pest species when crops belonging to the plant family Brassicaceae actually are associated with multiple pest species. In an attempt to address multiple pest species associated with Brassica crops, a trap crop mixture composed of mighty mustard (*Brassica juncea* [L.] Czern.), red Russian kale (*Brassica oleracea* L. var. acephala), and glossy collards (*Brassica oleracea* L. var. italica) (all Brassicaceae) was shown to be effective at attracting *P. xylostella*, imported cabbageworm (*Pieris rapae* [L.]) (Lepidoptera: Pieridae), cabbage aphid (*Brevicoryne brassicae* L.), and the harlequin bug (*Murgantia histrionica* [Hahn]) (Hemiptera: Pentatomidae), compared to the same species presented individually (R. Manandhar and J.C. Piñero, unpub. data). Once pests are concentrated on trap crops, they are commonly controlled by applying pesticides or by physical removal, but natural enemies likely play a role in suppression as well [1,22].

Insectary plants are a second habitat diversification tactic that can contribute to the regulation of insect pest densities. Insectary plants are flowering plants that are incorporated into agricultural ecosystems to attract and enhance the activity or density of beneficial arthropods on cash crops [1,11]. Insectary plants can increase the abundance or efficacy of natural enemies by providing alternative prey, pollen, nectar, and shelter [22,28,29]. For example, the longevity of parasitic wasps such as *Diadegma semiclausum* Hellén (Hymenoptera: Ichneumonidae) and *Cotesia glomerata* L. (Hymenoptera: Braconidae) increased when buckwheat, *Fagopyrum esculentum* Moench (Polygonaceae), and some species of Apiaceae were grown in a cabbage agro-ecosystem [30]. One challenge to this approach, however, is that the spillover of insectary-subsidized natural enemies into the crop habitat, and thus the spatial extent of biological control benefits, can be limited to the area in proximity to insectary habitats [31].

The roles of trap crops and insectary plants as biologically-based pest control methods are commonly studied independently, and their combined effects have not been investigated. Within an organic cabbage agro-ecosystem, the main goal of this study is to assess the effects of integrating the use of trap crops with insectary plants on the abundance of functionally important arthropod taxa represented by herbivores and their natural enemies. We predict that pairing trap crops with insectary plants will enhance their overall effectiveness: (1) trap crops will concentrate pests in an area adjacent to insectary plants where the spillover of natural enemies is high and (2) insectary-subsidised natural enemies will contribute to the natural suppression of pests within the trap crops, reducing the need for chemical control or physical removal.

## 2. Materials and Methods

### 2.1. Study Site

**Figure 1:**
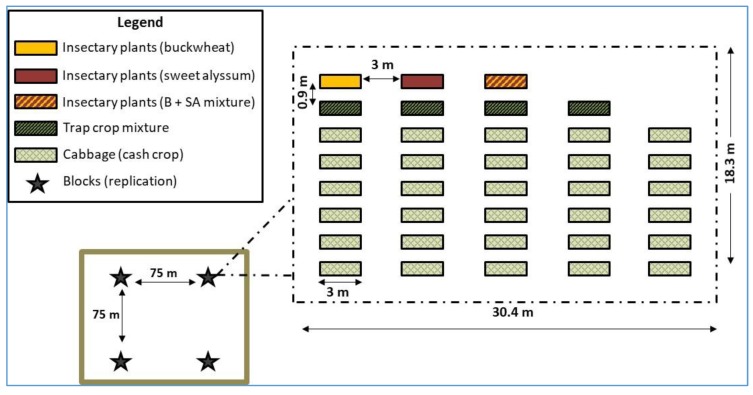
Diagrammatic representation of the field layout for the study conducted at the Lincoln University Alan T. Busby organic research farm. For each plot, the perimeter row was selected for insectary plants (buckwheat alone, sweet alyssum alone, and buckwheat in combination with sweet alyssum = B + SA), the second row was used for trap crop plants, and rows 3-8 were used for the cabbage cash crop.

This study was carried out from 29 June to 6 October 2016 at the Lincoln University Alan T. Busby organic research farm located in Jefferson City, Missouri, USA. The experimental area consisted of four 30.4 × 18.3 m blocks (replications) (Figure 1). Within each block, eight rows were prepared as raised beds that were 120 cm wide × 15.2 cm tall, and the distance between blocks was 75 m. The land was prepared on 13 May 2016 by plowing (about 20 cm deep), disking, and roto-tilling. Drip irrigation was installed on top of the beds, and they were covered with 1.2 m wide 1.0 mil white on black solid polyethylene mulch (Ginegar Plastics, Inc., Ginegar, Israel) for weed suppression. For each block, the perimeter row was used for insectary plants, the second row was used for trap crop plants, and rows 3–6 were used for the cabbage cash crop. Within each block, there were five plots (treatments, described below). Each plot was 3 m wide and separated from other plots by 3 m (Figure 1).

Every two weeks the grass was mowed (approx. 10 m) around experimental blocks using a flail-mower pulled by a tractor, and the soil was roto-tilled within each block to prevent weeds from interfering with the study. Finer trimming was conducted with a hand-held weed eater twice monthly. Supplemental irrigation was provided by drip tape from a gravity fed storage tank when there was not enough rain to meet crop needs. All plants were fertilized three times (on 26 July, and on 5 and 12 August) with a fish-based fertilizer (Organic Plant Nutrition 2-2-0, SF Organics, Thomson, IL, USA) listed by the Organic Materials Review Institute (OMRI). The OMRI-listed organic insecticide Azera™ (MGK, Minneapolis, MN, USA) was sprayed once, on 22 July 2016, at a rate of 70 mL/3.7 L only on the trap crop seedlings to ensure proper establishment due to risk posed by flea beetles. No other plants received insecticide, and no other pest management practices were undertaken.

### 2.2. Trap Crops, Insectary Plants, and Cabbage Cash Crop

The trap crop system evaluated consisted of a mixture of glossy collards (*B. oleracea* var. italica), kale (*B. oleracea* var. acephala), and mighty mustard (*B. juncea*), based on previous research conducted by R. Manandhar and J.C. Piñero (unpub. data). The two species of insectary plants evaluated were buckwheat (*F. esculentum*) (Polygonaceae) and sweet alyssum (*Lobularia maritima* (L.) Desv.) (Brassicaceae), either alone or in combination. These plants were selected based on previous research conducted by Lee and Heimpel [32], Lavandero et al. [33], Gillespie et al. [34], and Hogg et al. [35]. More recently, Shrestha [36] reported that both buckwheat and sweet alyssum performed best at attracting natural enemies under Missouri conditions. The cash crop was cabbage (*Brassica oleracea* var. capitata) cv. Golden Acre. All the seeds used for this study were organic and purchased from Johnny’s Selected Seeds (Winslow, ME, USA).

Five insectary plant/trap crop treatment combinations were evaluated: (1) buckwheat and trap crop mix; (2) sweet alyssum and trap crop mix; (3) buckwheat + sweet alyssum and trap crop mix; (4) trap crop mix without insectary plants; and (5) no insectary plants and no trap crops, i.e., cash crop alone (Figure 1). Trap crop species/insectary plant species treatment combinations were assigned randomly. For all plant species, seedlings were grown in pots with OM1 organic germinating media (Berger, Quebec, Canada) at the Lincoln University Dickinson Research Center greenhouse until transplant.

Trap crop and insectary seedlings were transplanted on 1 July 2016. The seedlings of buckwheat (3-week old) and sweet alyssum (5-week old) (14 plants of each species per treatment plot) were transplanted onto the perimeter row raised bed (=row 1) with 21.6 cm inter-plant spacing. Kale, glossy collards, and mighty mustard (6-week old) seedlings (three plants of each species per treatment plot) were transplanted with 46 cm inter-plant spacing. On 19 July 2016, 48 organic Golden Acre cabbage seedlings (6-week old) were transplanted onto the six raised beds (rows 3–8) per plot, at a density of eight plants per raised bed, with 46 cm inter-plant spacing.

### 2.3. Arthropod Sampling

Arthropods were monitored on insectary plants, trap crops, and the cash crop by visually inspecting plants during the months of August and September. Observations on insectary plants were conducted once a week, beginning when plants started flowering and ending when one of the species ceased to bloom. All observations were conducted from 9:00 to 11:00 a.m. on clear days. The upper third of each insectary plant in each plot was observed for a combined 5 min per plot. The number of arthropods visiting the target area was recorded, and all individuals were captured and identified to the family level [37] in the laboratory. A visit was recorded only if the arthropods landed on the plant.

The observations on trap crops were conducted twice a week from 4 August to 13 September 2016. Data included immature stages and adults of pests and natural enemies. All nine trap crop plants (three glossy collards, three red Russian kale, and three mighty mustard) were visually inspected in each treatment plot. The presence and abundance of economically important arthropod pests, which in this study were the cross-striped cabbageworm, *Evergestis rimosalis* (Guenée) (Lepidoptera: Crambidae); cabbage looper, *Trichoplusia ni* (Hübner) (Lepidoptera: Noctuidae); and the diamondback moth, *P. xylostella,* were recorded on each trap crop plant. Numbers of natural enemies, with a focus on predatory arthropods (adults and immature stages) and parasitoid cocoons, were also documented. Samples of parasitized cocoons were taken to the laboratory and incubated in cages until the emergence of adult Lepidoptera and/or parasitoids. Parasitoids were identified at the species level by Dr. Ben Puttler (Prof. Emeritus, University of Missouri, Columbia, MO, USA).

Similarly, a random sample of 18 cabbage cash crop plants from each replicated plot was thoroughly inspected, along with the abundance of selected arthropod pests and their natural enemies. This was done twice a week from 11 August to 20 September 2016. On 6 October 2016, 18 cabbage heads from each plot were harvested and weighed.

### 2.4. Statistical Analysis

*Insectary plant data:* The number of individuals of each of the four most abundant natural enemy families observed visiting insectary plants in 5-min sampling periods were compared across the three treatments where insectary plants were present (buckwheat alone, sweet alyssum alone, and a combination of both insectary plant species) by analysis of variance (ANOVA). Prior to analysis, the number of individuals of each insect family observed per plot was averaged across the eight sampling dates. *Trap crop data*: The number of individuals of each pest species (immature stages) and natural enemies (both adults and immature stages) observed on trap crop plants was compared across the four insectary plant treatment combinations where trap crops were present (no insectary plants, buckwheat alone, sweet alyssum alone, and a combination of both insectary plant species) by means of ANOVA. Analyses were conducted separately for kale and glossy collards using results from preliminary analyses indicating contrasting responses by natural enemies for each trap crop species and a very low response of the herbivore species prevalent in the study to mighty mustard. *Cash crop data*: Densities of herbivores on the cash crop were compared among trap crop species/insectary plant species treatment combinations using ANOVA.

For all analyses, means were separated, whenever appropriate, by Fisher-protected LSD tests at the 5% probability level. Whenever appropriate, data were transformed using √(x + 0.5) prior to analysis to stabilize variances. Statistical analyses were conducted in STATISTICA 13 [38]. All data are presented as the average number of insects per sampled plant, per sampling date.

## 3. Results

### 3.1. Herbivore Abundance on Trap Crops

The most abundant herbivore on trap crop plants was *E. rimosalis*, with mean densities of 18.2 ± 5.4 larvae per kale plant and 6.4 ± 2.5 larvae per collards plant, followed by *T. ni* (mean larval densities in kale and collards: 3.5 ± 1.8 and 1.7 ± 0.4 larvae, respectively). *Plutella xylostella* ranked third, with mean larval densities in kale and collards of 1.5 ± 0.3 and 0.8 ± 0.2, respectively. Herbivore densities recorded on mighty mustard (data not shown in Figures) were very low (0.23 ± 0.19, 0.08 ± 0.03, and 0.16 ± 0.09 for *E. rimosalis*, *T. ni*, and *P. xylostella*, respectively).

**Figure 2:**
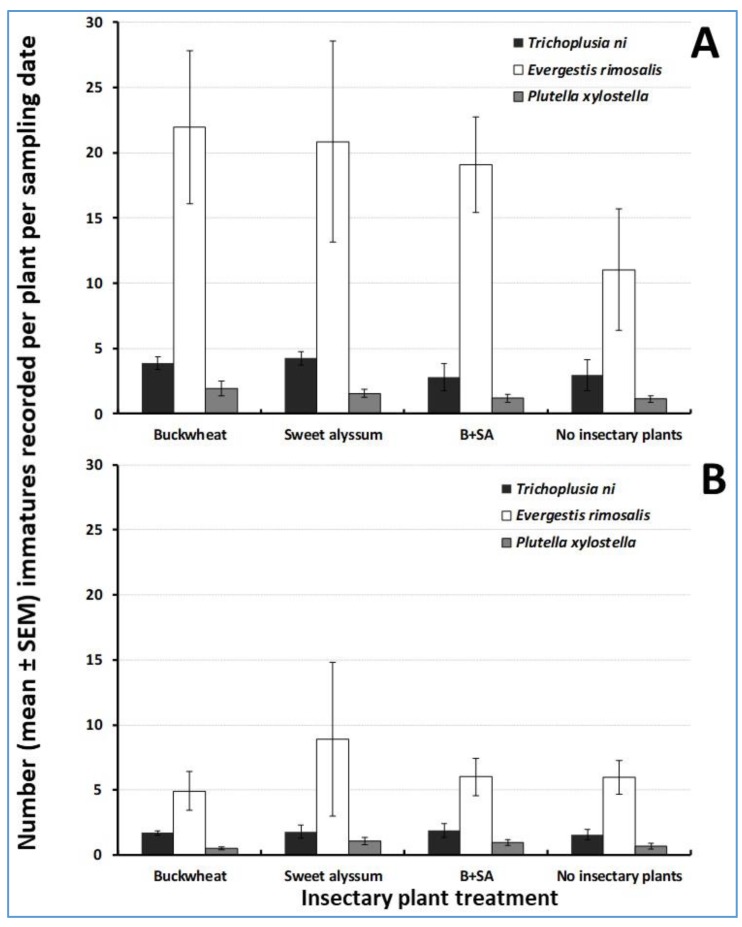
Trap crop herbivore abundance on a per-plant basis: **(A)** kale and **(B)** collards. For each herbivore species, there were no significant differences in their abundance on trap crops in response to insectary plant treatment according to ANOVAS and Fisher-protected LSD tests (*p* < 0.05).

Insectary plants did not influence significantly the larval abundance of any of the three herbivores (ANOVAS F_3, 12_ = 0.92, *p* = 0.46; F_3, 12_ = 1.07, *p* = 0.40; and F_3, 12_ = 0.98, *p* = 0.44 for *T. ni*, *E. rimosalis*, and *P. xylostella*, respectively) on kale (Figure 2A) and collards (ANOVAS F_3, 12_ = 0.08, *p* = 0.97; F_3, 12_ = 0.10, *p* = 0.96; and F_3, 12_ = 1.37, *p* = 0.30 for *T. ni*, *E. rimosalis*, and *P. xylostella*, respectively) (Figure 2B).

### 3.2. Natural Enemy Abundance on Trap Crops and Influence of Insectary Plants

**Figure 3:**
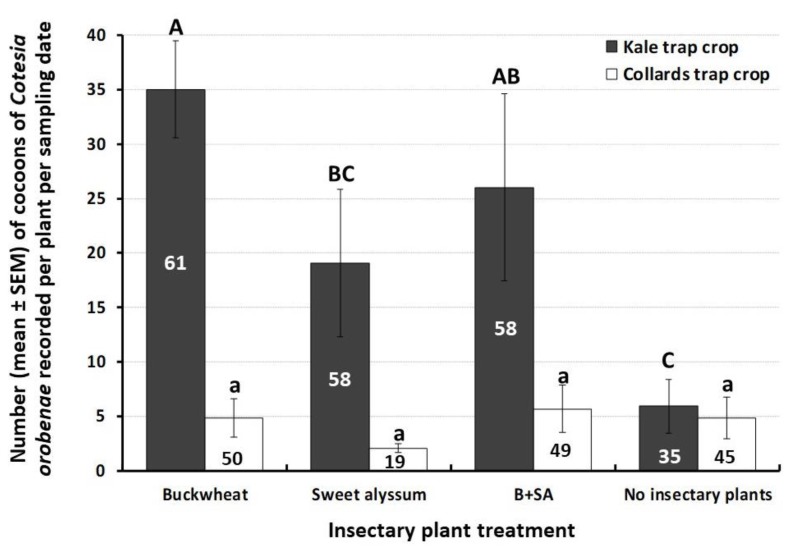
Trap crop parasitism of the herbivore *Evergestis rimosalis* by the braconid wasp *Cotesia orobenae*. B + SA = Buckwheat and sweet alyssum mixture. Numbers inside bars indicate average percent parasitism. For each trap crop species, bars with different letters are significantly different according to ANOVAS and Fisher-protected LSD tests (*p* < 0.05).

*Cotesia orobenae*, a gregarious endoparasitoid of *E. rimosalis*, was the most abundant natural enemy on trap crop plants. Irrespective of insectary plant treatment, the mean number of cocoons recorded was 21.5 ± 5.6 per kale plant and 4.4 ± 1.5 per collards plant. The level of parasitism (expressed as the number of cocoons of *C. orobenae*) of *E. rimosalis* caterpillars feeding on kale was affected significantly by insectary plant treatment. Significantly more parasitoid cocoons were recorded, on a per plant basis, on kale trap crops in the presence of buckwheat alone or the combination of buckwheat and sweet alyssum than when insectary plants were absent (ANOVA F_3, 12_ = 4.01, *p* = 0.03) (Figure 3). However, the level of parasitism of *E. rimosalis* by *C. orobenae* in collards was not influenced significantly by insectary plant species or arrangement (ANOVA F_3, 12_ = 0.51, *p* = 0.68) (Figure 3). The average percent parasitism recorded ranged from 19% to 61% (Figure 3).

**Figure 4:**
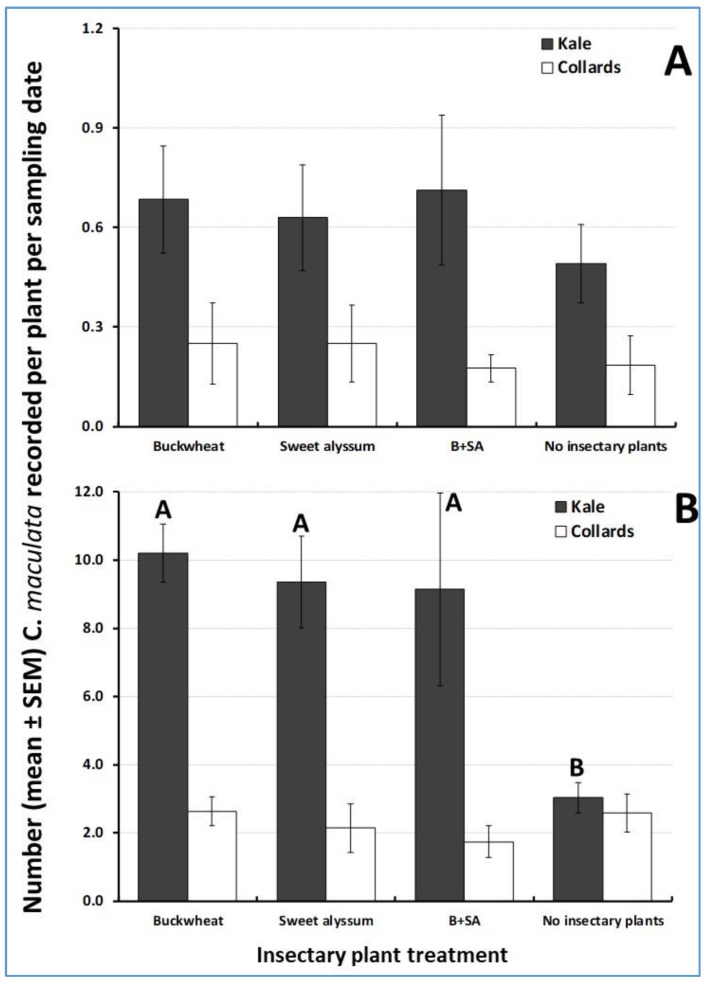
Trap crop pink lady beetle, *Coleomegilla maculata*, abundance on a per-plant basis. (**A**) adult stage, (**B**) immature stage (eggs and larvae combined). For each trap crop species, bars with different letters are significantly different according to ANOVAS and Fisher-protected LSD tests (*p* < 0.05).

The second most abundant natural enemy observed on trap crop plants was the predatory beetle *C. maculata.* Irrespective of insectary plant species, the mean number of adults and of immature stages recorded per sampling date was nearly three times greater for kale plant than for collards. The densities of *C. maculata* in the adult stage did not vary significantly across insectary plant treatments for kale (ANOVA F_3, 12_ = 0.31, *p* = 0.82) or collards (ANOVA F_3, 12_ = 0.06, *p* = 0.98) (Figure 4A,B). Significantly more immatures (eggs and larvae combined) were recorded on kale in the presence of insectary plants than in the absence of insectary plants, regardless of insectary plant identity (ANOVA F_3, 12_ = 5.36, *p* = 0.01) (Figure 4A). However, on collards, similar numbers of immature *C. maculata* were recorded regardless of the presence and type of insectary plants (ANOVA F_3, 12_ = 0.50, *p* = 0.69) (Figure 4B).

### 3.3. Attractiveness of Insectary Plants to Natural Enemies

A total of 347 individuals were recorded through visual observations at the peak flowering season of insectary plant species (Table 1). We observed a natural enemy community largely composed of three orders (Coleoptera, Diptera, and Hemiptera) and four families (Coccinellidae, Syrphidae, Tachinidae, and Geocoridae). Hymenopteran parasitoids were not recorded due to the difficulty associated with proper identification based on visual observations on site. Overall, buckwheat alone attracted the largest number of natural enemies, followed by the mixture of buckwheat and sweet alyssum. Sweet alyssum alone attracted the lowest number of natural enemies. The most abundant natural enemies recorded visiting insectary plants were *C. maculata* (43.5% of the total natural enemies observed) and syrphid flies (Diptera: Syrphidae) (42.4%) (Table 1).

**Figure 5:**
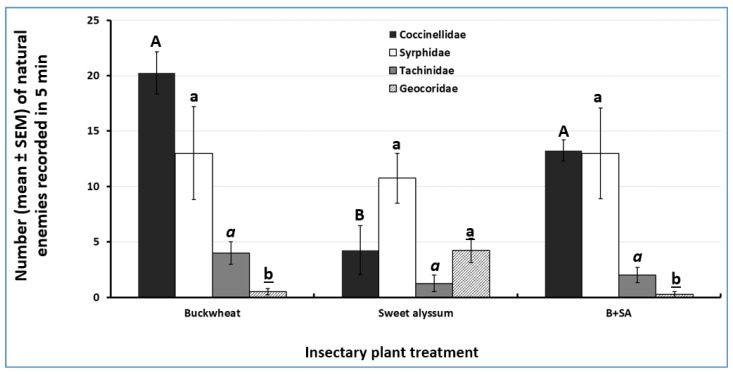
Insectary plant natural enemy abundance, on a per-plant basis. B+SA = Buckwheat and sweet alyssum mixture. For each insect family, bars with different letters are significantly different according to ANOVA and Fisher-protected LSD tests (*p* < 0.05).

Significantly more *C. maculata* adults were found on buckwheat alone than on sweet alyssum alone, whereas the mixture of both plants attracted an intermediate number of adults (ANOVA F_2, 9_ = 18.3, *p* < 0.001) (Figure 4). The number of syrphid flies (ANOVA F_2, 9_ = 0.05, *p* = 0.95) and tachinid flies (ANOVA F_2, 9_ = 3.20, *p* = 0.08) did not differ significantly across treatments. Sweet alyssum alone attracted significantly more big-eyed bugs (Hemiptera: Geocoridae) than the other two insectary plant treatments (ANOVA F_2, 9_= 14.7, *p* = 0.01) (Figure 5).

### 3.4. Herbivore Abundance in the Cabbage Cash Crop

**Figure 6:**
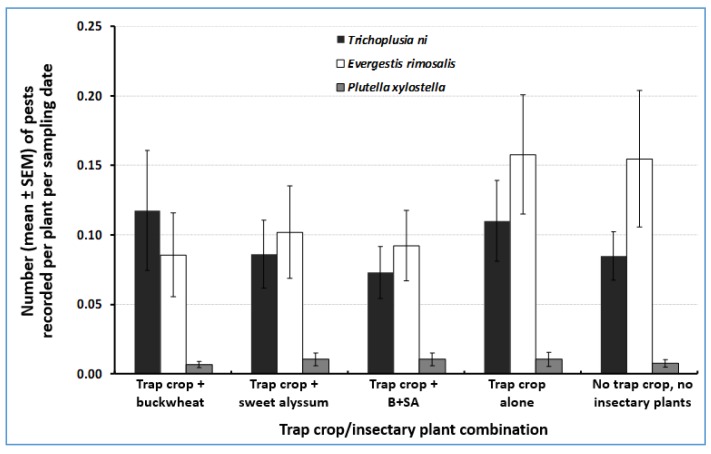
Pest cash crop abundance on a per-plant basis. B+SA= buckwheat + sweet alyssum. There were no significant differences in their abundance on cabbage plants in response to insectary plant treatment according to ANOVAS and Fisher-protected LSD tests (*p* < 0.05).

Overall, *E. rimosalis* was the most abundant herbivore on the cabbage cash crop, followed by *T. ni,* and *P. xylostella*. The abundance of pest species on the cash crop was low overall. For instance, on a per-plant basis, densities of the herbivores *E. rimosalis*, *T. ni*, and *P. xylostella* were 153.8, 36.9, and 161.4× greater on the kale trap crop than on the cabbage cash crop, and 54.4, 18.5, and 89× greater on the collards trap crop than on the cash crop. Herbivore abundance did not differ significantly across trap crop/insectary plant treatment combinations (ANOVA F_4, 114_ = 1.1, *p* = 0.35; F_4, 114_ = 1.0, *p* = 0.42; F_4, 114_ = 0.3, *p* = 0.88, for *E. rimosalis*, *T. ni*, and *P. xylostella*, respectively) (Figure 6).

### 3.5. Natural Enemy Abundance in the Cabbage Cash Crop

**Figure 7:**
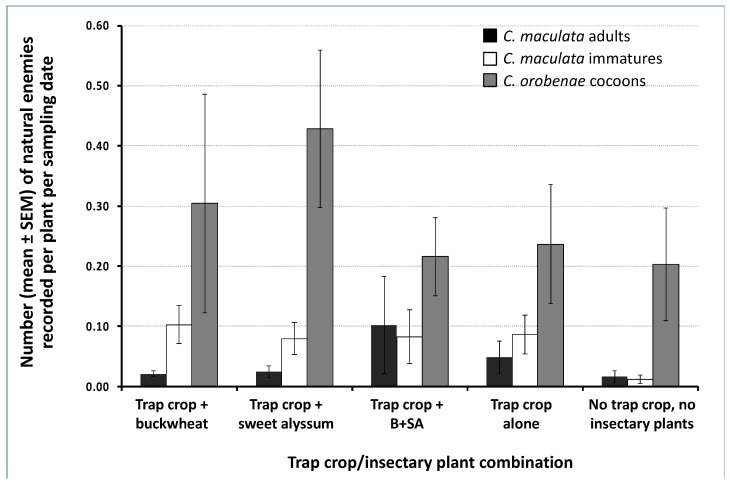
Natural enemy cash crop abundance on a per-plant basis. B+SA= buckwheat + sweet alyssum. There were no significant differences in their abundance on cabbage plants in response to insectary plant treatment according to ANOVAS and Fisher-protected LSD tests (*p* < 0.05).

Average densities of adults and immatures of *C. maculata* and immatures of *C. orobenae* on the cabbage cash crop were 0.04 ± 0.02, 0.07 ± 0.03, and 0.3 ± 0.1, respectively. No significant differences in mean densities of beneficials on the cash crop were found among trap crop/insectary plant treatments (ANOVA F_4, 15_ = 0.8, *p* = 0.53; F_4, 15_ = 1.3, *p* = 0.31; F_4, 15_ = 0.6, *p* = 0.68, for adult and immatures of *C. maculata* and immatures of *C. orobenae*, respectively) (Figure 7).

### 3.6. Cabbage Cash Crop Yield

Trap crops/insectary plant treatment combinations had no statistically significant treatment effects on cabbage yield (F_4, 98_ = 1.629, *p* = 0.173) (Table 2).

## 4. Discussion

This study revealed the potential of integrating trap crops with insectary plants as a way of concentrating pest herbivores in habitats where natural enemy activity is higher. Overall, buckwheat alone attracted the largest number of natural enemies, followed by the mixture of buckwheat and sweet alyssum. The presence of insectary plants significantly increased the abundance of immature stages of the predatory beetle *C. maculata* and parasitism of *E. rimosalis* by the gregarious endoparasitoid *C. orobenae* on trap crop plants compared to trap crops in the absence of insectary plants.

Trap crop plants were effective in concentrating herbivores, as hypothesized. For instance, average densities of *E. rimosalis*, *T. ni*, and *P. xylostella* were 154, 37, and 161× greater on the kale trap crop than on the cabbage cash crop, and 54, 18, and 89× greater on the collards trap crop than on the cash crop. Overall, kale was the most attractive trap crop to all herbivore species under the conditions of this study. For instance, kale concentrated more larvae of *E. rimosalis*, *T. ni*, and *P. xylostella*, respectively, than did glossy collards. While kale was the most effective trap crop plant in this study, the effectiveness of individual species can vary from one study to another. Studies conducted in Florida by Mitchell [39] reported that glossy collards are very effective trap crops for *P. xylostella*. However, in another study, collards did not successfully control *P. xylostella* in a commercial cabbage field in New York [24]. While in this study mighty mustard attracted very few herbivore species, previously this plant species performed well at attracting harlequin bugs and aphids (pests that were not present in the field throughout our study) (Piñero and Manandhar, unpub. data). Therefore, in the three-species trap crop mixture (glossy collards, kale, and mighty mustard), all three plants might provide insurance against variation in the pest community from year to year.

In the present study, the incorporation of insectary plants enhanced the activity of natural enemies in the trap crop. These findings highlight the role of trap crops in concentrating pests and their natural enemies near insectary plants. The most abundant natural enemy species observed in the adult stage of the insectary plant species evaluated here (buckwheat and sweet alyssum) was the pink lady beetle *C. maculata*. The second most abundant insect group observed was represented by flies in the family Syrphidae. The syrphid subfamily Syrphinae includes species that are predatory in the larval stage. However, the feeding habits of syrphid species are varied [40], and we did not identify individuals to the species level. Tachinid flies (Tachinidae) and big-eyed bugs (Geocoridae), two important natural enemy taxa, ranked third and fourth, respectively, in relative abundance. Previous studies have shown that buckwheat and sweet alyssum attract a diversity of natural enemies [12,41,42]. In this study, adult *C. maculata* were more frequently observed on buckwheat alone and on the mixture of buckwheat and sweet alyssum than on sweet alyssum alone. In contrast, big-eyed bugs were more attracted to sweet alyssum alone than to buckwheat and to the mixture of buckwheat and sweet alyssum. Factors explaining the seemingly reduced attractiveness of sweet alyssum when mixed with buckwheat to big-eyed bugs are unknown.

As is commonly the case, we found that natural enemies from insectary plants did not move out far into the cash crop, as denoted by the overall low densities of natural enemies recorded on cash crops, which also had lower pest densities (Figure 7). Blaauw and Isaacs [31] found no difference in the densities of natural enemies observed in blueberry crop fields with or without flower treatments. In our study, although no statistical differences were detected across treatments, *E. rimosalis* larvae were found in higher numbers on the Kale trap crop when adjacent to insectary plants (Figure 2). The effect of greater diversity of natural enemies in areas with greater diversity has been documented [43]. For example, in Philippine rice fields greater numbers of adult yellow stemborers, *Scirpophaga incertulas* (Lepidoptera: Pyrausdtidae), and parasitism by *Trichogramma japonicum* have been documented in diversified farming landscapes compared to conventional rice fields [44], which coincides with our results ( Figure 2; Figure 3). The higher number of *E. rimosalis* on trap crops adjacent to insectary plants could be due to the nectar availability for adult moths. For example, sweet alyssum appears to be highly attractive to *P. xylostella* adults, possibly for feeding purposes (on floral nectar), as survival of *P. xylostella* larvae developing on sweet alyssum is low [43].

An important contribution of this study is the documented increase in abundance of the parasitic wasp *C. orobenae* (as documented in terms of parasitoid cocoons recorded on *E. rimosalis*) and the predatory beetle *C. maculata* (as documented in terms of densities of immature stages) on the kale trap crop when in association with insectary plants. Such effects were independent from the densities of herbivores. *Cotesia orobenae* is a specialist larval endoparasitoid of the cross-striped cabbageworm and is gregarious in nature. The mean brood size cocoon masses vary from 8–12 cocoons per mass [45]. While this parasitic wasp is an effective natural enemy that provides excellent control of *E. rimosalis* in several regions of the USA, continuous use of insecticides to control *E. rimosalis* and other lepidopteran larvae such as *P. xylostella* [24] has disrupted populations of *C. orobenae*, causing localized outbreaks of *E. rimosalis* [46]. In our study, conducted in a certified organic farm, the high concentrations of host larvae as well as the presence of insectary plants may have helped to maintain populations of *C. orobenae* in the agro-ecosystem. We suggest that as long as this parasitic wasp is found in nature in adequate numbers, less pesticide would be required to control *E. rimosalis*. *Coleomegilla maculata* is an aphidophagous predator widely distributed in natural and managed ecosystems in North, Central, and South America [47]. In the present study, no aphids were present on any Brassica species; therefore, it was assumed that the main prey species of *C. maculata* were the eggs and larval stages of the main herbivores, *E. rimosalis*, *T. ni*, and *P. xylostella*.

Our study demonstrated complementarity in use of trap crops and insectary plants to concentrate pest herbivores in an area where natural enemy activity was enhanced. The most common practices used to lower pest densities on trap crops are by destroying plants by physical means or applying insecticides directly to trap crops [2,22,24]. For example, Blue Hubbard squash is an effective trap crop, which, when treated with insecticides, has successfully reduced densities of the striped cucumber beetle, *Acalymma vittatum* (Coleoptera: Chrysomelidae), by 94% in butternut squash, compared with conventional control methods [48]. Similarly, the use of mungbean, *Vigna radiates* (Fabaceae), as a trap crop to control *Apolygus lucoru* (Hemiptera: Miridae) in Bt cotton resulted in a 70% reduction in insecticides used [49]. The above studies used insecticides to kill pests on trap crops. In contrast, our study relied upon the activity of natural enemies to manage pests on trap crops. While we did not find statistical differences in pest densities in the cash crop across treatments, nor did we find significant differences in yield, we observed a greater trend towards higher yield in plots where trap crops were integrated with buckwheat insectary plants than in cash crop plots without trap crops or insectary plants. Such lack of detectable effects may have been the result of a strong response of herbivores to trap crop plants, which likely pulled herbivores away from control plots. An additional possible shortcoming could be the comparatively short distance between treatments. While some trap crop studies have successfully documented the accumulation of pests on trap crop plants [21,22,50], designing and conducting manipulative studies with trap crops at the proper scale has proven difficult [51]. Additionally, it is important to understand the migration pattern of the targeted insect pests and behavior of its natural enemies while considering using both trap crops and insectary plants as a reliable strategy for growers who are interested in biological management programs.

## 5. Conclusions

Our findings showed that in an organic cabbage system, trap crops pulled pests away from the cash crop, and insectary plants enhanced the abundance of key natural enemies (*C. maculata and C. orobenae*). This information increases our understanding of how habitat complexity within the farm can impact the relationship between agricultural pests and their natural enemies. The ‘*Botanical Triad*’ of cash crop, trap crop, and insectary plants represents a new type of agro-ecosystem manipulation that integrates ecosystem service providers (represented in this study by predators and parasitoids) with the cropping system. Botanical triads are expected to contribute to enhancing and conserving functional biodiversity in cropped areas through ecological stacking, aka ‘ecostacking’ [52], via the integration of ecosystem service providers with the rest of the cropping system in support of more sustainable vegetable production in organic systems.

## Figures and Tables

**Table 1:** Number of natural enemy individuals visiting insectary plant species recorded during visual observations according to insectary plant species and arrangement.

Natural Enemy Family (# Species Recorded. ND = Not Determined)	Buckwheat	Mixture of Buckwheat and Sweet Alyssum	Sweet Alyssum	Total	% of Total
Coccinellidae (1)	81	53	17	151	43.5
Syrphidae (ND)	52	52	43	147	42.4
Tachinidae (ND)	16	8	5	29	8.3
Geocoridae (1)	2	1	17	20	5.8
Total	151	114	82	347	100.0

**Table 2:** Cabbage yield, expressed as mean weight of 18 cabbage heads sampled per plot, according to the trap crop/insectary plant treatment combinations.

Trap Crop–Insectary Plant Combination	Yield (Grams) Means ± SEM
Trap crop present—buckwheat	1072.50 ± 131.10
Trap crop present—sweet alyssum	747.25 ± 88.23
Trap crop present—buckwheat and sweet alyssum	982.50 ± 89.30
Trap crop present—no insectary plants	995.25 ± 110.71
No trap crop—no insectary plants	811.09 ± 85.03
